# Level of medication adherence and its determinants of cardiovascular disease patients attending at specialized teaching hospitals of Amhara regional state, Ethiopia: a multicenter cross-sectional study

**DOI:** 10.3389/fphar.2024.1422703

**Published:** 2024-07-30

**Authors:** Samuel Berihun Dagnew, Samuel Agegnew Wondm, Fisseha Nigussie Dagnew, Yohannes Shumet Yimer, Yehualashet Teshome Wondmkun, Tilaye Arega Moges

**Affiliations:** ^1^ Clinical Pharmacy Unit, School of Pharmacy, College Health Sciences, Debre Tabor University, Debre Tabor, Ethiopia; ^2^ Clinical Pharmacy Unit, School of Pharmacy, College of Medicine and Health Sciences, Debre Markos University, Debre Markos, Ethiopia; ^3^ Social and Administrative Pharmacy Unit, School of Pharmacy, College Health Sciences, Debre Tabor University, Debre Tabor, Ethiopia; ^4^ Department of Pharmacy, College of Health Sciences, Debre Birhan University, Debre Birhan, Ethiopia

**Keywords:** adherence, cardiovascular, determinate factors, Ethiopia, ordinal logistic regression

## Abstract

**Background:**

Non-adherence to medication in patients with cardiovascular disease continues to be a main cause of suboptimal management, increased morbidity and mortality, and increased healthcare expenses. The present study assessed the level of medication adherence and its determinants of cardiovascular disease patients.

**Methods:**

An institutional-based multicenter cross-sectional study was conducted with patients with cardiovascular disease in Northwest Ethiopian teaching hospitals. The level of medication adherence was evaluated using a standardized questionnaire of the Adherence in Chronic Disease Scale (ACDS). To find determinants of the level of medication adherence, an ordinal logistic regression model was employed. Statistics were significant when P ≤ 0.05 at a 95% confidence interval (CI).

**Results:**

In the end, 336 participants were included in the research. According to this study, one-third of patients had low medication adherence, half had medium adherence, and one-fifth had high medication adherence. Elderly patients [adjusted odds ratio (AOR) = 2.691; 95% confidence interval (CI), 1.704–4.251; P < 0.000], marital status (AOR = 1.921; 95% CI, 1.214–3.039; P = 0.005), alcoholic patients (AOR = 2.782; 95% CI, 1.745–4.435; P < 0.000), Patients without physical activity (AOR = 1.987; 95% CI 1.251–3.156; P = 0.004), non health insurances (AOR = 1.593; 95% CI 1.003–2.529; P = 0.049), sever Charles comorbidity index (AOR = 2.486; 95% CI 1.103–5.604; P = 0.028), patients with polypharmacy (AOR = 2.998 (1.817–4.947) P < 0.000) and, manypolypharmacy (AOR = 3.031 (1.331–6.898) P = 0.008) were more likely to have low medication adherence.

**Conclusion:**

The current study concluded that one-third of study participants had low medication adherence. Older age, marital status, drinker, physical inactivity, drug source, comorbidity, and polypharmacy all contributed to the low level of medication adherence. To improve patients with cardiovascular disease’s adherence to their medications, intervention is necessary.

## 1 Introduction

Patients with multiple morbidities and chronic non-communicable diseases have increased consistently globally in particular, Cardiovascular disease (Bennett et al., 2018; Burnier, 2023). Cardiovascular diseases (CVDs) are the primary cause of death. Over 75% of CVD-related deaths occur in nations with lower and moderate incomes (Korkmaz and CARDIOVASCULAR DISEASES AND PREVENTION, 2021; Rehman et al., 2021). One of the main factors influencing good treatment outcomes is patient medication nonadherence. The majority of cardiovascular patients have been found they couldn’t recall their medications (Kravitz et al., 1993). Nonadhernace with prescribed treatment is a severe issue that impacts the patient as well as the healthcare system (Jimmy and Jose, 2011; McKenzie et al., 2015). Medication adherence refers to how often a person follows health practitioners’ recommendations whether it comes to drug use or altering their lifestyle (Sabaté, 2003). Numerous chronic drugs are frequently administered to people with cardiovascular disease. Furthermore, regimens have become increasingly complex (Shalansky and Levy, 2002). Medication complexity, which includes the number of prescription drugs supplied, their dosage forms, frequency of dosing, and instruction for using is the major factor for non-adherence.

Nonadherence to medication is a worldwide issue that jeopardizes people’s health and economic results for society (Stewart et al., 2023). Thus, patients are linked to worse treatment results, illness progression, and estimated billions of dollars worth of preventable direct medical expenses annually (DiMatteo, 2004a; DiMatteo, 2004b; Fuchs, 2022). Numerous studies have shown that poorer drug adherence worsened health consequences that extend beyond insufficient treatment of clinical parameters (Sokol et al., 2005; Perreault et al., 2012; Kim et al., 2018). Nevertheless, a lack of adherence has a detrimental impact on many other areas of managing diseases, such as disease exacerbations that occasionally result in acute hospitalizations, higher health costs, and, of course, a decline in the quality of life for patients (Kim et al., 2018; Peacock et al., 2021). An increased average length of hospital stay, functional impairments, poor quality of life, frequent hospital and pharmacy visits, and the occurrence of some more recent health problems or diseases can all be caused by inadequate adherence to the medication (K et al., 2023).

Analyzing the factors that influence adherence could result in improved treatment and results. Because adherence is a multi-factor phenomenon that depends on several factors rather than just one, factors impacting treatment adherence are frequently complex (Kolandaivelu et al., 2014). However, marital status, monthly income, level of education, drug regimen, and co-morbidities are the elements that influence the development of medication non-adherence. It becomes more expensive for the government and people to provide adequate healthcare in a developing nation (Saeed et al., 2023).

Understanding what obstacles patients face to taking their medications as prescribed is essential to improving medication adherence. Developing successful adherence-enhancing therapies requires a deeper knowledge of the factors that lead to poor drug adherence (Van der Laan et al., 2019; Nakajima et al., 2021). This study assessed the level of medication adherence and its determinants of cardiovascular disease patients attending Amhara Regional Teaching Hospitals.

## 2 Methods and materials

### 2.1 Study setting, design, and period

The research was carried out in the Amhara region of Ethiopia from July 1 to 30 September 2023, at three teaching hospitals: Tibebe Ghion, University of Gondar, and Debre Birhan (Hakim Gizaw). The northern and northwest regions of Ethiopia are home to the Amhara region. There are 181 woredas, or districts, 78 urban centers, and 10 administrative zones. The University of Gondar, Tibebe Ghion, and Debre Birhan (Hakim Gizaw) are the three teaching hospitals in the region. More than 15 million patients have received care annually from the studied hospitals.

A multicenter cross-sectional study was conducted in teaching hospitals in the Amahara region to assess the level of adherence among patients with cardiovascular disease and the factors associated with non-adherence between July 1 and 30 September 2023, gregorian calendar.

### 2.2 Study population and sampling technique

Patients who visited the University of Gonder, Debre Birhan (Hakim Gizaw) hospitals, and Tibebe Gion Teaching Hospital for medication follow-ups at chronic disease clinics encompassed the source population. The study population comprises all cardiovascular patients who attended treatment during the data collection period. Using a single population proportion formula $n=\frac{\;{\left(Z\alpha /2\right)}^{2}P\left(1-P\right)}{d^{2}}$ , where n = sample size required, d = is the margin of error 5% (d = 0.05), Z = the degree of accuracy required (95% level of significance = 1.96), and P = the proportion of nonadherence in patients with cardiovascular disease, assumed to be 0.5 (50%). n = 1.962 0.5 (1–0.5)/0.052 = 384.16 = −384. This was determined by taking the 50% proportion of treatment non-adherence for major cardiovascular in concurrent cases and for associated factors (since the investigators are not aware of any similar study in Ethiopia). The total number of cardiovascular patients in hospitals is 1510. The final sample size (NF) was determined using a correction procedure because the research population is fewer than 10,000. If NF = n/1 + n/N, then 384/1 + 384/1510 = 336. Up until the needed sample was reached, all patients with cardiovascular illness who met the inclusion criteria and showed up for follow-up during the data-collecting periods were achieved. In the end, 134, 121, and 81 suitable patients were included in the chronic disease follow-up clinics of Tibebe Goin, University of Gonder, and Debre Birhan (Hakim Gizaw) hospitals, respectively, in proportion to the number of patients with cardiovascular disease in the hospitals. Using a consecutive sampling technique, study participants from three chosen hospitals were enrolled.

### 2.3 Eligibility criteria

All patients who were 18 years of age or older, who visited the chronic disease clinic for follow-up, whose charts plainly showed a diagnosis of cardiovascular diseases, and who were receiving treatment during follow-up were included in the study. Patients from the settings’ data over the preceding 3 months follow-up were eligible for this study. Cardiovascular diseases are collectively referred to as heart and blood vessel illnesses which include coronary heart disease, cerebrovascular disease, peripheral arterial disease, rheumatic heart disease, congenital heart disease, deep vein thrombosis, and pulmonary embolism disorders (Nangia et al., 2016). Conversely, patients who did not voluntarily communicate were excluded from the research.

### 2.4 Data collection instruments, procedures, and quality control

A structured questionnaire was used as a data-gathering tool using journals that were previewed (Nangia et al., 2016; Kubica et al., 2017; Van der Laan et al., 2019; Peacock et al., 2021; Fuchs, 2022). The data collection instrument was created in English after a review of other related studies on related subjects, with minor adjustments made on account of the local clinical surroundings. The sociodemographic information about the participants, including gender, age, place of residence, religion, ethnicity, income, marital status, level of education, BMI, residency, educational status, employment status, physical activity, and health insurance status were included.

Following instructions regarding the study’s objectives, data collection tools and producers, and ethical considerations, three hospital-based pharmacists gathered the data. The participants in the data collection were willing. Primary data came from in-person patient interviews, and medical records from the patients were used to document laboratory results, medical conditions, and prescription dosages.

Based on several relevant investigations, a systematic questionnaire was created. Monitoring and verifying that all surveys were completed ensured the quality of the data. Three professionals reviewed the questionnaire’s face validity to ensure that the questions were clear. After that, 10% of research participants not included in the final analysis had the survey pretested for content, design, readability, and comprehension. Next, a suitable modification was implemented. Following a 2-day training session, three pharmacists collected the data. The study’s goal as well as the methods and instruments used for gathering data were made very clear by the supervisor. Using the WHO’s recommendations, socio-cultural adaptation was carried out, and adjustments were made in response to feedback. As a result, the survey yielded accurate results and was simple to comprehend and complete.

### 2.5 Outcome measurement

#### 2.5.1 Adherence measurement

The Adherence for Chronic Disease (ACDs) which was validated by Kubica, A., et al., in 2017 was used for assessing adherence. This scale consists of seven items, each of which has five possible alternatives. The questions address both the situations and viewpoints that may have an indirect impact on adherence (Questions 6 and 7) as well as the behaviors that directly determine adherence (Questions 1–5). Every question has five possible answers, A through E. A stands for four, B for three, C for two, D for one, and E for zero points. The results fall between 0 and 28 points: a total score of less than 21 indicates low adherence, a total score of 21–26 indicates medium adherence and a total score of more than 26 indicates high adherence (Kubica et al., 2017).

### 2.6 Data Processing and analysis

Every piece of information gathered was personally examined for consistency and completeness of response. Data were cleaned, coded, and then imported into EpiData version 4.6 before being exported to STATA for additional analysis. To investigate the data’s normal distribution, Shapiro-Wilk tests, Q-Q plots, and histograms were employed. Using descriptive statistics, means (±SD), proportions, tables, and figures, the characteristics of the study participants were outlined, and the study findings were presented. The relationship between the level of medication adherence and other predictor variables was examined using an ordinal logistic regression model. The statistical techniques of taste of parallel line and the proportional odds assumption were applied to all variables and those that met the assumptions of ordinal logistic regression. To discover predictor variables with the level of medication adherence, variables with *p* < 0.25 in the bivariate analysis were taken into consideration for additional analyses in the multivariable analysis. The significance level was *p* ≤ 0.05 at 95% CI.

### 2.7 Ethical approval and participants’ consent

The Debre Tabor University Ethical Review Committee gave its ethical approval to the proposal with a reference number of CHS/265/2023. The research subjects were required to give their informed consent after being fully informed about the goals of the investigation. Written informed consent was acquired from every person in the study who was included. The study followed Helsinki legislation in terms of doing it in a properly anonymous and confidential manner.

## 3 Results

### 3.1 Sociodemographic characteristics of the study participants

The research had 336 study participants in total. The participants’ average age (±SD) was 63.06 ± 13.45 years, with 57.34% of them being females. A BMI of less than 25 was found in 79.76% of the patients. About half of patients (53.27%) get their medication for free. Of the subjects, one-fifth (20.24%) acquired adverse drug reactions (Table 1).

**TABLE 1:** Sociodemographic characteristics of cardiovascular disease patients at teaching hospitals in Amahara region, Ethiopia, 2023 (N = 336).

Variables	Categories	Frequency	Percentage	Mean (SD)
Gender	Male	143	42.56	
	Female	193	57.44	
Age	<65	165	49.11	63.06 ± 13.45
	≥65	171	50.89	
Body mass index (kg/m2)	<24.9	268	79.76	22.67 ± 2.97
	≥25	68	20.24	
Marital status	Married	173	51.49	
	Single/others	163	48.51	
Level of educations	Less than college	301	89.58	
	College and above	35	10.42	
Occupational status	Farmer	224	66.67	
	Merchant	62	18.45	
	Government employee	11	3.27	
	Retire	39	11.61	
Religions	Orthodox	270	80.36	
	Muslim	56	16.67	
	Protestants	7	2.08	
	Others	3	0.90	
Monthly income (ETB)	<5,000	142	42.26	5,075.8 (±1,832.1)
	≥5000	194	57.74	
Current alcohol intake	Yes	186	55.36	
	No	150	44.64	
Residence	Urban	105	31.25	
	Rural	231	68.75	
Physical activities	Yes	134	39.88	
	No	202	60.12	
Health insurance	Yes	179	53.27	
	No	157	46.73	
Adverse drug reactions	Yes	68	20.24	
	No	268	79.76	

NB: ETB, ethiopian birr.

### 3.2 Disease characteristics of the study participant

Based on the Charles comorbidity index level, nearly half (48.21%) of the patients’ diseases were mild, according to the study. The most common diagnoses for the patients were heart failure (38.39%), hypertension (36.31%), and stroke (33.93%) (Table 2).

**TABLE 2:** Diseases pattern of cardiovascular disease patients at teaching hospitals in Amahara region, Ethiopia, 2023.

Types of diseases		Frequency	Percentage	Mean (SD)
Heart failure		129	38.39	
Hypertension		122	36.31	
Stroke		114	33.93	
Coronal artery diseases		69	20.54	
Dyslipidemia		66	19.64	
Valvular heart disease		45	13.39	
Cor pulmonale		29	8.63	
Rheumatic heart disease		21	6.25	
Venous thrombus embolism		18	5.36	
Atrial fibrillation		17	5.06	
Peripheral artery disease		4	1.19	
CCI	Mild	162	48.21	2.74 ± 1.15
	Moderate	145	43.15	
	Sever	29	8.63	

CCI, charles comorbidity index.

### 3.3 Medications profile of the study participants

In this investigation, the majority of patients (45.83%) were treated with diuretics, followed by ACEIs/ARBs (36.01%), and dyslipidemia (31.25%), and mean (SD) of the drugs 5.69 ± 2.43 Table 3

**TABLE 3:** Medication profiles of cardiovascular disease patients at teaching hospitals in Amahara region, Ethiopia, 2023.

Class of medications		Frequency	Percentage	Mean (SD)
Diuretics		154	45.83	
ACEIs/ARBs		121	36.01	
Antidyslipidemia		105	31.25	
Antiplatelet		93	27.68	
Calcium channel blockers		81	24.11	
Anticoagulants		50	14.88	
Beta-blockers		41	12.20	
Digitals		28	8.33	
Vasodilators		19	5.65	
Others*		20	5.95	
Number of drugs	<5	115	34.23	5.69 ± 2.43
	5–9	187	55.65	
	>9	34	10.12	

NB: ACEIs/ARBs: Angiotensin Converting Enzyme Inhibitors & Angiotensin Receptor Blockers, Others*: Antifungal (2), Phosphodiesterase (2), Anticonvulsant (4), Anthelmintics (1), Antiviral (2), B. agonist (5), Antithyroid (4).

### 3.4 Level of medication adherence of the study participants

**FIGURE 1:**
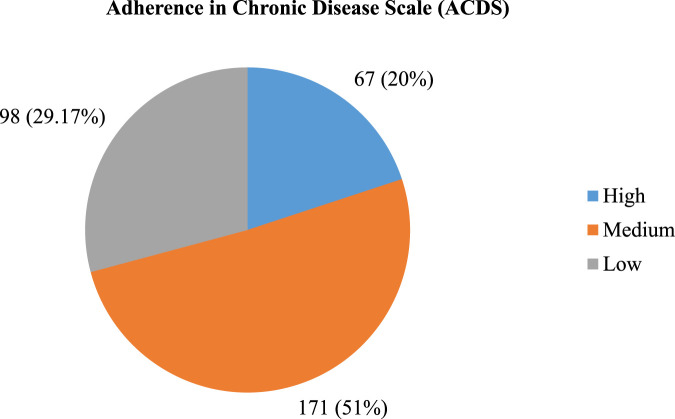
Level of adherence among cardiovascular disease patients at teaching hospitals in Amahara region, Ethiopia, 2023 (N = 336).

According to the Adherence in Chronic Disease Scale (ACDS), the mean and standard deviation of the adherences was 22.73 ± 4.49. Nearly one-third (29.17%) of patients had low adherence, and approximately half (50.89%) of research participants had medium adherence (Figure 1).

### 3.5 Interventions for low adherence of the patients

Based on the analysis of all low adherence cases, 92.86% of them had intervations; this means that 62.25% of patient counseling, 13.27% of spoken information to family members or caregivers, and 9.18% of written information was given (Table 4)

**TABLE 4:** Interventions given for low adherence among cardiovascular disease patients at teaching hospitals in Amahara region, Ethiopia, 2023 (98).

Innervations	Specific intervention given	Frequency percentage	
Interventions given	Patient (drug) counseling	61	62.25
	Spoken to family member/caregiver	13	13.27
	Written information provided (only)	9	9.18
	The patient referred to the prescriber	6	6.12
	Other intervention (specify)	2	2.04
No interventions given		7	7.14

### 3.6 Sociodemographic determinants of medication adherence

Ordinal logistic regression analysis was used to identify predictor variables of the level of medication adherence. According to the results of the multivariable ordinal logistic regression analysis, the level of medication adherence was substantially correlated with older age, marital status, alcoholism, lack of physical activity, and drug source. Comparing elderly people to adults, the lower level of adherence has increased by 2.7 times (AOR = 2.691; 95% CI, 1.704–4.251; *p* < 0.000). (AOR = 1.921; 95% CI, 1.214–3.039; *p* = 0.005) shows that the likelihood of singles and other nonmarried individuals had 1.9 times lower levels of adherence than married individuals. Compared to non-drinkers, patients who had consumed alcohol had 2.8 times higher odds of having poor adherence (AOR = 2.782; 95% CI, 1.745–4.435; *p* < 0.000). Patients without physical activity were twice more likely to have low levels of adherence than those with regular physical activity (AOR = 1.987; 95% CI 1.251–3.156; *p* = 0.004). When compared to patients who receive their medication for free, medication by payment adherence is significantly worse by the odds of 1.6 times (AOR = 1.593; 95% CI 1.003–2.529; *p* = 0.049) (Table 5).

**TABLE 5:** Independent factors for the low level of medication adherence of cardiovascular disease patients at teaching hospitals in Amahara region, Ethiopia, 2023 (N = 336).

Variables	Categories	Level of adherences			OR	*p*-value	AOR	*p*-value
		Low	Medium	High				
Gender	Male	32	81	30	1		1	
	Female	66	90	37	1.469 (0.974–2.216)	0.066	1.188 (0.763–1.850)	0.444
Age	<65	24	96	45	1		1	
	≥65	74	75	22	3.532 (2.287–5.455)	0.000	2.691 (1.704–4.251)	**0.000**
BMI	<24.9	80	141	47	1		1	
	≥25	18	30	20	1.518 (0.905–2.545)	0.113	1.642 (0.848–3.179)	0.142
Marital status	Married	30	96	47	1		1	
	Single/others	68	75	20	3.099 (2.018–4.758)	0.000	1.921 (1.214–3.039)	**0.005**
Level of educations	Less than college	91	157	53	2.545 (1.275–5.079)	0.008	1.716 (0.785–3.754)	0.176
	College and above	2	14	14	1		1	
Monthly income	<5,000	28	76	38	1		1	
	≥5000	70	95	29	2.197 (1.442–3.348)	0.000	1.531 (0.972–2.412)	0.066
Current alcohol intake	Yes	77	86	23	3.634 (2.344–5.632)	0.000	2.782 (1.745–4.435)	**0.000**
	No	21	85	44	1		1	
Physical activities	Yes	24	69	41	1		1	
	No	74	102	26	2.803 (1.816–4.325)		1.987 (1.251–3.156)	**0.004**
Residence	Urban	71	121	39	1		1	
	Rural	27	50	28	1.494 (0.959–2.327)	0.076	0.995 (0.552–1.795)	0.987
Health insurance	No	32	108	39	2.275 (1.495–3.462)	0.000	1.593 (1.003–2.529)	**0.049**
	Yes	66	63	28	1		1	
ADRs	Yes	32	27	9	2.459 (1.459–4.146)	0.001	1.499 (0.838–2.681)	0.172
	No	66	144	58	1			

NB: BMI, body mass index; ADRs, Adverse Drug Reactions; COR, crude odd ratio; AOR, adjusted odd ratio; CI, confidence interval, *p*-value ≤0:05, ∗*p*-value.

Bold values repersent statistical significance.

### 3.7 Diseases determinants of medication adherence

Regarding the disease patterns that determine the adherence level, the Charles Comorbidities Index and stroke have a substantial correlation with the adherence level. Accordingly, the likelihood of a patient experiencing a stroke increases low adherence levels by a factor of 2.7 (AOR = 2.729; 95% CI 1.732–4.3011; *p* < 000). When compared to patients with mild CCI, those with severe CCI exhibited chances of a 2.5 higher probability of low-level adherences (AOR = 2.486; 95% CI 1.103–5.604; *p* = 0.028) (Table 6).

**TABLE 6:** Diseases factors for the low level of medication adherence of cardiovascular disease patients at teaching hospitals in Amahara region, Ethiopia, 2023 (N = 336).

Variables	Categories	Level of adherences			OR	*p*-value	AOR	*p*-value
		Low	Medium	High				
Stroke	Yes	46	55	13	2.295 (1.481–3.557)		2.729 (1.732–4.3011)	**0.000**
	No	52	116	54	1		1	
AF	Yes	6	6	5	2.467 (0.729–8.345)	0.146	3.078 (0.889–10.658)	0.076
	No	92	165	62	1		1	
RHD	Yes	8	7	6	2.188 (0.853–5.614)	0.103	2.496 (0.939–6.636)	0.067
	No	90	164	65	1		1	
CCI	Mild	44	76	42	1		1	
	Moderate	45	81	19	1.407 (0.920–2.151)	0.115	1.467 (0.948–2.269)	0.085
	Sever	9	14	6	2.263 (1.024–5.001)	0.044	2.486 (1.103–5.604)	**0.028**

**NB**: CCI, charles comorbidity index; AF, atrial fibrillation; RHD, rheumatic heart disease; COR, crude odd ratio; AOR, adjusted odd ratio; CI, confidence interval; *p*-value ≤0:05, ∗*p*-value.

Bold values repersent statistical significance.

### 3.8 Medication determinants of medication adherence

Medication patterns that were found to have an impact on medication adherence include the number of medications used, diuretics, CCBs, and anticoagulants. Compared to individuals who did not take diuretics, diuretic users had lower levels of adherence [AOR = 1.730, 95% CI (1.094–2.736); *p* = 0.019]. Patients taking CCBs were more likely to have low levels of adherence than those not taking the medicine by odds of 2.2 [AOR = 2. 153, 95% CI (1.116–3.129); *p* = 0.017]. AOR = 1.642, 95% CI (1.025–2.629); *p* = 0.039] shows that patients on anticoagulants had a 1.6-fold higher risk of low adherence than those not on these medicines. Similarly, patients who had more than five medications (AOR = 2.998 (1.817–4.947) *p* < 0.000) and patients who had more than nine medications (AOR = 3.031 (1.331–6.898) *p* = 0.008) were also more likely to have low medication adherence (Table 7).

**TABLE 7:** Medication factors for the low level of medication adherence of cardiovascular disease patients at teaching hospitals in Amahara region, Ethiopia, 2023 (N = 336).

Variables	Categ ories	Level of adherences			COR	*p*-value	AOR	*p*-value
		Low	Medium	High				
ACIs/ARBs	Yes	18	24	8	1.421 (0.802–2.517)	0.229	0.727 (0.391–1.351)	0.314
	No	80	147	59	1		1	
CCBs	Yes	35	34	12	2.076 (1.276–3.376)	0.003	2. 153 (1.116–3.129)	**0.017**
	No	63	137	55	1		1	
Antiplatelet	Yes	38	44	11	2.142 (1.351–3.397)	0.001	1.262 (0.632–2.518)	0.510
	No	60	127	56	1		1	
Diuretics	Yes	53	75	26	1.530 (1.015–2.306)	0.042	1.730 (1.094–2.736)	**0.019**
	No	45	96	41	1		1	
Statins	Yes	45	49	11	2.577 (1.642–4.043)	0.000	1.787 (0.896–3.565)	0.099
	No	53	122	56	1		1	
Beta-blockers	Yes	16	19	6	1.620 (0.868–3.025)	0.129	1.032 (0.532–2.001)	0.926
	No	82	152	61	1		1	
Anticoagulants	Yes	42	66	13	1.845 (1.206–2.821)	0.005	1.642 (1.025–2.629)	**0.039**
	No	56	105	54	1		1	
Number of medications	<5	13	64	38	1		1	
	5–9	72	92	23	3.836 (2.398–6.138)	0.000	2.998 (1.817–4.947)	**0.000**
	>9	13	15	6	4.239 (1.959–9.175)	0.000	3.031 (1.331–6.898)	**0.008**

NB: ACEIs/ARBs, Angiotensin Converting Enzyme Inhibitors & Angiotensin Receptor Blockers; CCBs, Calcium Chanal Blockers; COR, crude odd ratio; AOR, adjusted odd ratio; CI, confidence interval; *p*-value ≤0:05, ∗*p*-value.

Bold values repersent statistical significance.

## 4 Discussion

This study revealed the level of medication adherence among patients with cardiovascular diseases attending teaching hospitals in the Amahara region by using an ordinal logistic regression model. Globally, cardiovascular disease is the primary cause of death. The most difficult task in healthcare practice continues to be ensuring medication adherence in patients with chronic diseases, particularly in multimorbid patients, due to the complexity and many burdens of medications.

The current study found that half of the patients had medium adherence and one-third had poor adherence. Compared to studies from India, this study found relatively lower levels of adherence, with 44.4% of elderly patients with both diabetes and hypertension non-adherence (Jeyalakshmi et al., 2023). However, a study carried out in Jordan reveals that low (27.7%), medium (22.9%), and high (49.4%) (Al-Tarawneh et al., 2023). Another study in Taiwan found that low and high medication adherences were (34%) and medium adherences (32%) (Tsai et al., 2012). The variations in findings amongst studies may be due to various approaches, populations, economic conditions, and medication adherence instruments.

The results of this study indicated that non-adherence was more common in people over 65 years. This study’s findings were corroborated by some research including people with chronic disease (Mazzaglia et al., 2009; Jimmy and Jose, 2011; Kim et al., 2019; Li et al., 2021; Al-Tarawneh et al., 2023). There are numerous explanations for non-adherence in the specific group of older individuals. Among these include a higher likelihood of drug-related side effects as a result of pharmacodynamic and pharmacokinetic alterations, as well as a high frequency of comorbidity leading to polypharmacy.

In our study, patients who were single or otherwise unmarried had twice the chance of having poor adherence than patients who were married. This is consistent with a North Carolina study that found that single people had poor adherence rates twice (Trivedi et al., 2008; Wu et al., 2012; Doggrell and Kairuz, 2014). The reason behind married patients’ higher adherence rate compared to single patients remains unknown. Researchers have hypothesized that one explanation would be that spouses encourage adherence by offering helpful assistance (Kulkarni et al., 2006).

Alcohol use is a reliable indicator of poor treatment outcomes due to a decline in their cognitive abilities and unhealthy consequences may result from medication non-adherence. According to the current study, patients who were alcoholics had 2.8 times the likelihood of having low probability adherence. This result is in line with a study conducted in China (Li et al., 2021) that outlined the increased risk of medication nonadherence in patients who drank alcoholic drinks. Our finding is also partly consistent with those of Ahmed and colleagues, who discovered a link between alcohol use and a lack of adherence to diabetic self-care practice (Ahmed et al., 2006), non-adherence was associated with alcohol consumption (Aminde et al., 2019), Medication adherence was found to be negatively impacted by alcohol use, according to a systematic study (Grodensky et al., 2012). In another study conducted in Korea compared to people who never drank, moderate and heavy drinkers were less likely to exhibit good medication adherence (Han et al., 2017). However, the frequency of hospital visits by patients is negatively correlated with alcohol usage, according to prior research (Armstrong et al., 1998).

Patients in this trial who did not engage in physical activity had a two times higher likelihood of nonadherence than those who did. From a public health standpoint, physical activity can enhance wellbeing, medication adherence, and quality of life while lowering the burden of disease on both patients and the medical system (Kallings, 2008). Regular physical exercise has a good impact on medication adherence, according to a study done on hypertension patients at a cardiology unit in Italy (Sampogna et al., 2023). Another study conducted in Southwest Missouri State proves that exercise has a positive impact on improving medication adherence (Dishman, 1982).

Our findings show that patients who have no health insurance receiving their medication had nonadherence 1.6 times more frequently than those receiving it for free. This study’s supporting literature suggests that a rise in medicine nonadherence related to cost may be caused by growing consumer goods prices and drug costs (Dusetzina et al., 2023). One of the biggest obstacles to medication adherence is the financial strain and expense of prescription drugs (Chen et al., 2014). Another study also showed that sources of medications have a greater impact on medication adherence (Gast and Mathes, 2019). Medical costs associated with non-adherence may increase, as well as the frequency of doctor visits and hospital stays. The annual cost of healthcare could be decreased by addressing drug adherence.

Regarding the disease factors, patients with stroke would experience a 2.7-fold increase in low adherence. Because of polypharmacy and multimorbidity, stroke patients are more likely to have lower levels of medication adherence and self-care behaviors. Given the correlation between stroke and a roughly two-fold increased risk of cognitive decline, a stroke would be a significant contributing factor to nonadherence (Rohde et al., 2017). Consistent with what we discovered Numerous research suggest that stroke is the main reason for nonadherence (Crayton et al., 2018; Liu et al., 2021; Basheti et al., 2022; Cao et al., 2022).

The majority of research indicated that medication adherence was significantly associated with comorbidities. Patients with cardiovascular disease with comorbidities are especially difficult to adhere to their medication regimens. Patients in our study who had severe comorbidities exhibited 2.5 times lower levels of adherence than those with less severe comorbidities. Medication adherence for co-occurring disorders can be extremely challenging for individuals with cardiovascular illness, and non-adherence could hurt overall health. This has been documented in several studies that have demonstrated the influence of comorbidities on medication nonadherence (Loeppke et al., 2011; Curkendall et al., 2013; Smaje et al., 2018; Doya et al., 2024).

Identifying nonadherence in every patient who is not responding to treatment is the most crucial step toward increasing adherence because medication non-adherence and cardiovascular illness are expected to rise in frequency (Nelson et al., 2024). Thus, low adherence was more common in our study when patients were using calcium channel blockers, diuretics, and anticoagulants. Consistent with our findings, different studies discovered that the pharmacological class most strongly linked to nonadherence was CCBs, diuretics, and BBs (Kronish et al., 2011; Lemstra and Alsabbagh, 2014). However, some earlier research indicates that patients with diuretics had low adherence (Suol Thanh et al., 2022). This is due to Patients may skip dosages or restrict their medicine due to high expenses or difficulties getting refills of some medications; Patients who have uncomfortable or irritating side effects from their medications may purposefully miss doses or stop taking them completely; Complicated dosage regimens, multiple daily dosages, and the requirement to take the medication with food or on an empty stomach might make it difficult for patients to remember and follow the prescribed course of action.

Patients with cardiovascular illness are more likely to be polypharmacy, which means that it is associated with non-adherence. The occurrence of low adherence rates was significantly linked in our study to patients with polypharmacy and multiple polypharmacy, as these patients are less likely to take their medications as prescribed. On top of this, due to the increased number of prescriptions that can be overlooked daily, polypharmacy can result in medication nonadherence. Comparably, several drugs have been linked to medication adherence in the literature (Smaje et al., 2018; Wakai et al., 2021; Cao et al., 2022). Several studies also confirm that drug adherence has been published, with polypharmacy being one of the most often researched risk factors for nonadherence (Cooney and Pascuzzi, 2009; Nakajima et al., 2021; Basheti et al., 2022).

Clinical pharmacists can improve treatment outcomes for patients by improving patient adherence and managing cardiovascular diseases. In our study, 92.86% of nonadherence had interventions. For cardiovascular disease patients to have optimal treatment outcomes, it is imperative to identify and resolve adherence hurdles such as drug cost, transportation concerns, and healthcare availability. Medication non-adherence has a negative impact on psychosocial variables, expenses, and results that are shared by patients, caregivers, healthcare systems, payers, and society at large (Bosworth et al., 2017).

### 4.1 Strength and limitation

The study was strong in several areas. Because it was a multicenter study, its generalizability was enhanced. The study site had enough resources because it was a teaching Hospital. The study also establishes a correlation between subjective and objective findings. Furthermore, a consecutive sampling approach was employed. This might have lessened the study’s source of bias. The present research has certain shortcomings. Patients’ self-reported adherence measuring scale, which is based on respondents’ honesty and faith in them, is used to estimate the adherence level. Responses may be influenced, leading to an overestimation or underestimation of the medication adherence level. Notwithstanding these drawbacks, the power of our investigation was sufficient, and the results aligned favorably with those of other global studies of a comparable nature.

## 5 Conclusion

Cardiovascular drug adherence lowers morbidity and death significantly while also saving money on medical expenses. Medication nonadherence persists despite these benefits because of several obstacles at the patient, provider, and systemic levels. The current study concluded that one-third of study participants had low medication adherence, roughly half had medium adherence, and one-fifth had good adherence. Older age, marital status, drinker, physical inactivity, drug source, comorbidity, and polypharmacy all contributed to the low level of medication adherence. Therefore, to slow the course of the disease and achieve better treatment outcomes, healthcare professionals should endeavor to increase patient knowledge of the importance of taking their medication adherence.

## Data Availability

The raw data supporting the conclusions of this article will be made available by the authors, without undue reservation.
