# Supplementary material for: Level of medication adherence and its determinants of cardiovascular disease patients attending at specialized teaching hospitals of Amhara regional state, Ethiopia: a multicenter cross-sectional study
Source: Front Pharmacol. 2024 Jul 30;15:1422703. doi: 10.3389/fphar.2024.1422703 (PMC11319153; doi:10.3389/fphar.2024.1422703)
Supplement: Supplementary file 1 [file DataSheet1.PDF]

Annex (Questionnaire)

Written informed consent:

Participant information sheet and consent form (English version)

Dear respondent my name is ________________________________ I am here to collect data for a study entitled "Level of Medication Adherence and its Determinants of Cardiovascular Disease Patients Attending at Specialized Teaching Hospitals of Amhara Regional State by Using Ordinal Logistic Regression Model: A Multicenter Cross-Sectional Study". The principal investigator is Mr. Samuel Berihun who is a lecturer of clinical pharmacy in Debere Tabor University, College of Health Sciences, and School of Pharmacy. The purpose of this study is to assess level of medication adherence and its determinants of cardiovascular disease patients attending at specialized teaching hospitals. To achieve the study objective, your honest and genuine participation by responding to the question prepared is very important and highly appreciated. You will not receive any payment or other personal benefit for participation in this study. But study findings will be used to better improve patient care in the study setting. There is also no cost to you for participation but it will take 10-15 minutes of your time. Your participation in this study is completely voluntary. You have the right to be in the study or to withdraw in any time; there is no influence that insists you to participate, unless you voluntarily confirmed to participate. Your refusal to participate will in no way affect your service at the hospital. We would like to assure you that privacy will strictly be maintained throughout. Your personal information will be maintained through the use of unique identifiers.

Are you willing to participate in this program? A. yes B. no

Signature ____________________

If yes, continue the interview

General information

Code ____________________

Date______________________

Part I. Socio-demographic characteristics /make tick to answer the following questions.

SECTION 1: Sociodemographic demographic characteristics

1. Age (years) …………………………………………………………..
2. Height……………………weight………………BMI………………
3. Gender: A. Female B. Male
4. Religion: A. Muslim B. Orthodox C. Protestant D. Others (specify)…..
5. Current marital status
   - A. Single C. Married
   - B. Widowed D. Divorced E. Separated
6. Level of education
   - A. can't read and write C. primary education
   - B. secondary education D. higher education
7. Job/Occupation:
   - A. Office work C. Farmer E. Merchant
   - B. Student D. Daily laborer F. Others, specify____________
8. Physical exercise A. yes B. No
9. Residence: A. Rural B. Urban
10. Herbal medicine use: A. Yes B. No
11. Alcohol use: A. alcoholic B. Non alcoholic
12. Smoker: A. smoker B. non-smoker
13. Source of medicine: A. Payment B. Free
14. Self-management of medication: A. self B. Relatives
15. Physical activity A. yes B. no
16. ADR A. yes B. no
17. Monthly income (ETB) ____________________

Part II Medical and medication characteristics

1. Chief compliant………………………………………………………………………
2. HPI………………………………………………………………………………………
   ………………………………………………………………………………………………
3. Past Medication History

4. MainAssessment………………………………………………………………………………
………………………………………………………………………………………………

5. Current medication

No	Start date	Stop date	Medication regimen (dose, Route, Frequency)	Class of medication

6. Number of medications………………………………………...
7. Charlson comorbidity index (CCI)……………………………….
8. Duration disease in year………………………………………..

Part III: Laboratory Related data

No	Tests	Parameters	Value
1.	Renal function test	Scr.	
		BUN	
2	Liver Function test (LFT)	SGOT	
		SGPT	
3	Lipid panel (mg/dL)	LDL-C	
		HDL-C	
		TC	
		TG	
4	Coagulation profile	INR	
		PT	
		APTT	
5.	CBC	WBC	
		Neutrophil	
		PLT	
6.	Cardiac biomarkers	Troponins	
		CK-MB	
7.	Glucose value	HbA1C	
		FBS	
		RBS	
8.		BP	

The Adherence in Chronic Diseases Scale (ACDS)

1. Do you always remember to take all your medications according to your doctor’s instructions?

A. Always B. Almost always C. Sometimes D. Hardly ever E. Never

2. Do you happen to change the dosing of your medications without prior consultation with your doctor?

A. Never B. Only occasionally C. Sometimes D. Frequently

E. I do not adhere to my doctor’s recommendations at all

3. Do you adjust the dosing of your medications according to how you feel?

A. No, I strictly follow the prescribed dosing, no matter how I feel

B. Yes, I reduce the dosage of some medications when I feel good

C. Yes, I skip doses of some medications when I feel good

D. Yes, I temporarily discontinue some medications when I feel good

E. Yes, I discontinue all medications when I feel good

4. On the appearance of medication-related side effects (e.g., stomach pain, liver pain, rash, lack of appetite, oedema):

A. I seek medical attention instantly

B. I reduce the dosage of the medication and attempt to expedite the elective appointment with my doctor

C. I discontinue the medication and attempt to expedite the elective appointment with my doctor

D. I discontinue the medication and wait for the next elective appointment with my doctor

E. I discontinue all my medications and wait for the next elective appointment with my doctor

5. Do you find all your medications necessary for your health?

A. Yes, I do B. I find most of my medications to be beneficial for my health

C. I find only some of my medications to be beneficial for my health

D. I find some of my medications to be beneficial for my health, while the others to be harmful for me

E. I find the majority of my long-term medications to be harmful for me

6. Does your doctor inquire about medication-related problems that you might possibly experience?

A. Yes, on every appointment B. Yes, he/she usually does C. Yes, but only sometimes

D. Yes, but only occasionally E. No, never

7. Do you tell truth when asked by your doctor about medication-related problems?

A. Yes, always B. Almost always

C. I try to be honest, but sometimes it is hard to admit to non-compliance with doctor's recommendations

D. Sometimes yes, another time no

E. No, I don't. I find it my own private business

Results are within the range of 0–28 points

Score: A — 4 B — 3 C — 2 D — 1 E — 0

Total score < 21 points Low adherence

Total score 21–26 points medium adherence

Total score > 26 points High adherence

Part V: Interventions

**Innervations**	**Specific intervention given**	**Tick**
Interventions given	Patient (drug) counseling	
	Spoken to family member/caregiver	
	Written information provided (only)	
	The patient referred to the prescriber	
	Other intervention (specify)	
No interventions given		

ትርጉም ጥያቄዎች

የአሳታፊ መረጃ ወረቀት እና የፍቃድ ቅጽ (የእንግሊዘኛ ቅጂ)

ውድ ምላሽ ሰጪ ስሜ ________________________________ እባላለው" የመድኃኒት ተገዢነት ደረጃ እና የልብና የደም ቧንቧ ሕሙማን ቆራጥነት በአማራ ክልል ስፔሻላይዝድ ማስተማሪያ ሆስፒታሎች የሚከታተሉት መደበኛ ሎጂስቲክስ ሪግሬሽን ሞዴል፡ ባለብዙ ማዕከል አቋራጭ ጥናት"፡፡ በተመለከተ መረጃ ለመሰብሰብ እዚህ መጥቻለሁ። ዋና መርማሪው አቶ ሳሙኤል በሪሁን በደበረታቦር ዩኒቨርሲቲ ጤና ሳይንስ ኮሌጅ የፋርማሲ ትምህርት ቤት የክሊኒካል ፋርማሲ መምህር ናቸው። የዚህ ጥናት አላማ የመድኃኒት ተገዢነት ደረጃ እና የልብና የደም ቧንቧ ሕሙማን ቆራጥነት በአማራ ክልል ስፔሻላይዝድ ማስተማሪያ ሆስፒታሎች የሚከታተሉት መደበኛ ሎጂስቲክስ ሪግሬሽን ሞዴል፡ ባለብዙ ማዕከል አቋራጭ ጥናት ። ከዚህ ጥናት የተገኙ ውጤቶች የሕክምና እርካታ ደረጃን, የመድሃኒት ስህተቶችን እና በእሱ ላይ ተጽዕኖ ሊያሳርፉ የሚችሉ ተለዋዋጭዎችን ለማወቅ ጠቃሚ ናቸው. የጥናት አላማውን ለማሳካት ለተዘጋጀው ጥያቄ ምላሽ በመስጠት በታማኝነት እና በእውነተኛ ተሳትፎዎ በጣም አስፈላጊ እና በጣም የተመሰገነ ነው። በዚህ ጥናት ውስጥ ለመሳተፍ ምንም አይነት ክፍያ ወይም ሌላ የግል ጥቅም አያገኙም። ነገር ግን የጥናት ግኝቶች በጥናቱ መቼት ውስጥ የታካሚ እንክብካቤን በተሻለ ሁኔታ ለማሻሻል ጥቅም ላይ ይውላሉ. እንዲሁም ለመሳተፍ ምንም አይነት ወጪ የለዎትም ነገር ግን ጊዜዎን ከ10-15 ደቂቃዎች ይወስዳል። በዚህ ጥናት ውስጥ ያለዎት ተሳትፎ ሙሉ በሙሉ በፈቃደኝነት ነው። በጥናቱ ውስጥ የመሳተፍ ወይም በማንኛውም ጊዜ የመተው መብት አለዎት; ለመሳተፍ በፈቃደኝነት እስካልተረጋገጠ ድረስ እርስዎ እንዲሳተፉ የሚያስገድድ ምንም ተጽእኖ የለም። ለመሳተፍ ፈቃደኛ አለመሆንዎ በምንም መልኩ በሆስፒታሉ ውስጥ ያለውን አገልግሎት አይጎዳውም. ግላዊነት በጥብቅ እንደሚጠበቅ ልናረጋግጥልዎ እንወዳለን። የግል መረጃዎ ልዩ መለያዎችን በመጠቀም ይቀመጣል። ፈቃደኛ ነወት፡

ሀ እሽ ለ. እምቢ

ፊርማ __________________

ክፍል I. ማህበራዊ-ስነ-ህዝብ ባህሪዎች፤ የሚከተሉትን ጥያቄዎች ለመመለስ / ምልክት ያድርጉ ።

1. ዕድሜ (ዓመት) _________________

2. ፆታ፡   ወንድ   ሴት

3. ክብደት (ኪግ) ___ ቁመት (ሴ.ሜ) ___ የሰውነት ብዛት ማውጫ (ቢኤምአይ) [ኪግ / ሜ 2]

____

4. የጋብቻ ሁኔታ፡ ያላገባ/ች   ያገባ/ች   የፈታ/ች የሞተበት/ባት

5. የትምህርት ደረጃ፡ ያልተማረ/ች   የመጀመሪያ ደረጃ   ሁለተኛ ደረጃ   ኮሌጅ እና ከዚያ በላይ

6. ሃይማኖት፡ ኦርቶዶክስ ሙስሊም ፕሮቴስታንት አድቬንቲስት ሌሎች_________________

7. የሙያ ደረጃ፡ ሀ. ገበሬ ለ. ነጋዴ ሐ. የመንግስት ሰራተኛ መ. ጡረታ የወጣ/ች

8. እንቅስቃሴ ያረጋሉ   ሀ. አው   ለ. አላደረግም

9. የመኖሪያ ቦታ፡ ሀ. የከተማ   ለ. ገጠር

10. አልኮል መጠቀም፡- ሀ. በጭራሽ   ለ. በመደበኛነት

11. አጨሽ፡ ሀ. አጨሽ   ለ. የማያጨስ

12. የመድኃኒት ምንጭ ፡   ሀ. ክፍያ   ለ. ነፃ

13. መድሃኒት እራስን ማስተዳደር   ሀ. እራስ   ለ. በአተሰብ

“ACDS” መድኃኒትን በታዘዘው መሰረት በአግባቡ ስለመውሰድ” መለኪያ

1. በሐኪምዎ መመሪያ መሠረት ሁሉንም መድሃኒቶችዎን መውሰድዎን ሁልጊዜ ያስታውሳሉ?

ሀ) ሁል ጊዜ ለ) ሁል ጊዜ ማለት ይቻላል ሐ) አንዳንድ ጊዜ መ) በጭራሽ በጭራሽ ሠ) በጭራሽ

2. ከሐኪምዎ ጋር ያለ ቅድመ ምክክር ያለዎትን የመድኃኒት መጠን ልክ ይለውጣሉ?

ሀ) በጭራሽ ለ) አልፎ አልፎ ሐ) ብቻ አንዳንድ ጊዜ መ) በተደጋጋሚ ሠ) በጭራሽ የዶክተሮቼን ምክሮች አላከብርም

3. በሚሰማዎት ስሜት መሠረት የመድኃኒቶችዎን ልክ መጠን ያስተካክላሉ?

ሀ) አይ ፣ እኔ ምንም ያህል ቢሰማኝም የታዘዘልኝን መድሃኒት በጥብቅ እከተላለሁ

ለ) አዎ ፣ ጥሩ ስሜት ሲሰማኝ የአንዳንድ መድኃኒቶችን መጠን እቀንሳለሁ

ሐ) አዎ ፣ ጥሩ ስሜት ሲሰማኝ የአንዳንድ መድኃኒቶችን መጠን እዘላለሁ

መ) አዎ ጥሩ ስሜት ሲሰማኝ አንዳንድ መድሃኒቶችን ለጊዜው አቋርጣለሁ

ሠ) አዎ ፣ ጥሩ ስሜት ሲሰማኝ ሁሉንም መድሃኒቶች አቋርጣለሁ

4. ከመድኃኒት ጋር የተዛመዱ የጎንዮሽ ጉዳቶች (ለምሳሌ የሆድ ህመም ፣ የጉበት ህመም ፣ ሽፍታ ፣ የምግብ ፍላጎት እጥረት ፣

እብጠት)

ሀ) ወዲያውኑ የሕክምና እርዳታ እፈልጋለሁ

ለ) የመድኃኒቱን መጠን እቀንሳለሁ እና ከሐኪሜ ጋር የተመረጠውን ቀጠሮ ለማፋጠን እሞክራለሁ

ሐ) መድሃኒቱን አቋርጣለሁ እና ከሐኪሜ ጋር የተመረጠውን ቀጠሮ ለማፋጠን እሞክራለሁ

መ) መድሃኒቱን አቋርጬ ከሐኪሜ ጋር የሚቀጥለውን የምርጫ ቀጠሮ እጠብቃለሁ

ሠ) ሁሉንም መድኃኒቶቼን አቋርጬ ከሐኪሜ ጋር የሚቀጥለውን የምርጫ ቀጠሮ እጠብቃለሁ

5. ሁሉንም መድሃኒቶችዎን ለጤንነትዎ አስፈላጊ ሆነው ያገ Doቸዋል?

ሀ) አዎ፣ በሚገባ ለ) አብዛኞቹ መድኃኒቶቼ ለጤንነቴ ጠቃሚ ሆነው አግኝቻቸዋለሁ

ሐ) ለጤንነቴ ጠቃሚ የሆኑ አንዳንድ መድኃኒቶቼን ብቻ አገኛለሁ

መ) አንዳንድ መድኃኒቶቼ ለጤንነቴ ጠቃሚ ሲሆኑ ሌሎቹ ደግሞ ለእኔ ጎጂ ሆነው አግኝቸዋለሁ

ሠ) አብዛኛዎቹ የረጅም ጊዜ መድኃኒቶቼ ለእኔ ጎጂ ሆነው አግኝቸዋለሁ

6. ሐኪምዎን ምናልባት ሊያጋጥሙዎት ከሚችሉት መድኃኒቶች ጋር ስለሚዛመዱ ችግሮች ይጠይቃል?

ሀ) አዎ ፣ በእያንዳንዱ ቀጠሮ ላይ ለ) አዎ ፣ እሱ / እሷ ብዙውን ጊዜ ይነገረኛል ሐ) አዎ፣ ግን አንዳንድ ጊዜ ብቻ

መ) አዎ ፣ ግን አልፎ አልፎ ብቻ ነው ሠ) አይ ፣ በጭራሽ

7. ከመድኃኒት ጋር ስለሚዛመዱ ችግሮች በሐኪምዎ ሲጠየቁ እውነቱን ይናገራሉ?

ሀ) አዎ ፣ ሁል ጊዜ ለ) ሁልጊዜ ማለት ይቻላል

ሐ) ሐቀኛ ለመሆን እሞክራለሁ ፣ ግን አንዳንድ ጊዜ የዶክተሮችን ምክሮች አለመከተል መቀበል በጣም ከባድ ነው

መ) አንዳንድ ጊዜ አዎ ፣ ሌላ ጊዜ የለም

ሠ) አይ, እኔ አላደርግም
